# John Cross, epidemic theory, and mathematically modeling the Norwich smallpox epidemic of 1819

**DOI:** 10.1371/journal.pone.0312744

**Published:** 2024-11-13

**Authors:** Connor D. Olson, Timothy C. Reluga

**Affiliations:** 1 Department of Mathematics, Penn State University, University Park, PA, United States of America; 2 Huck Institute of Life Sciences, Penn State University, University Park, PA, United States of America; Shanxi University, CHINA

## Abstract

In this paper, we reintroduce Dr. John Cross’ neglected and unusually complete historical data set describing a smallpox epidemic occurring in Norwich, England in 1819. We analyze this epidemic data in the context of early models of epidemic spread including the Farr–Evans–Brownlee Normal law, the Kermack–McKendrick square Hyperbolic Secant and SIR laws, along with the modern Volz–Miller random-network law. We show that Cross’ hypothesis of susceptible pool limitation is sufficient to explain the data under the SIR law, but requires parameter estimates differing from the modern understanding of smallpox epidemiology or large errors in Cross’ data collection. We hypothesize that these discrepancies are due to the mass-action hypothesis in SIR theory, rather than significant errors by Cross, and use Volz–Miller theory to support this. Our analysis demonstrates the difficulties arising in inference of attributes of the disease from death incidence data and how model hypotheses impact these inferences. Our study finds that, combined with Volz–Miller modeling theory, Cross’ death incidence data and population observations give smallpox attributes which largely cohere to those used in modern smallpox models.

## Introduction

While many scientists love in their hearts to feel themselves engaging in the perpetual accumulation of scientific knowledge, we must reluctantly admit that each piece of knowledge is a hard-won trophy of a mud-wrestling match, often fought as much with each other and ourselves as with data and facts. Not even the science of luminaries like Newton [1], Mendel [2], and Kepler [3] goes unblemished.

Mathematical epidemiology has its own example of mis-interpreted data. In their foundational papers (cited more than 10,000 times according to Google Scholar), Kermack and McKendrick [4] compared the results of the now well-known compartmental SIR model to data from a plague epidemic in Bombay (the colonial name for Mumbai) in 1906 [5]. This comparison “… implies that the rates did not vary during the period of the epidemic…” [4], and thus supported one of the central premises of the paper—that the course of an epidemic could be explained well by the partial but incomplete exhaustion of the supply of susceptible people, either by death or transformation through the acquisition of immunity.

But as Bacaër [6] pointed out, the 1906 epidemic was less straight-forward. The data Kermack and McKendrick employed was a count of spill-over cases (human infections from an animal-borne disease) from a seasonal cycle of plague incidence in mice and rats that had been on-going since at least 1897, and would continue beyond 1911 [6]. Thus, seasonal forcing provides at least a partial explanation for this incidence data. The seasonality seems to have entirely escaped mention by Kermack and McKendrick [4, 7–9]. So, despite Kermack and McKendrick’s assertions, the seasonality of plague incidence in mice and rats implies the 1906 epidemic wasn’t a novel epidemic in a population lacking immunity. Also, new cases were principally created by spill-over from mice and rats to humans via a flea vector rather than human-to-human transmission. Thus, in several aspects, the 1906 epidemic is contrary to fundamental assumptions of the SIR model Kermack and McKendrick present [4].

Although Kermack and McKendrick’s plague example was misleading, other datasets pre-dating their work were consistent with their hypothesis. The analysis of Brownlee [10] is notable in this regard, though he himself subscribed to an alternate hypothesis that epidemic limitation followed the exhaustion of the causative agent. William Farr’s data sets on smallpox [11] and “cattle plague” [12] may be appealed to as well. But perhaps the most well-described epidemic to which SIR theory may be constructively compared is a 1819 smallpox epidemic in Norwich, England, excellently documented by John Cross [13], later referenced by Creighton [14] in his 1894 history of British epidemics, and now easily accessible online.

Given how the fame of historical figures like John Snow has been at least partially a deliberate social construct [15, 16] rather than a true attribution of credit, our motivation for producing this paper is rooted in the historical significance of the dataset we draw from, along with it’s pedagogical value. Cross [13] was potentially the first person to conjecture that the termination of the epidemic was due to a decrease in the number susceptible individuals. We contend that the data set he provides is a succinct historical example of epidemic termination by such an exhaustion. With the revelation of the inadequacy of the Bombay epidemic example [6], and human contact patterns of the 1820’s being potentially more consistent with the law of mass-action’s strong-mixing hypothesis, we campaign for the use of Cross’ work to fill the void as the canonical example of a simple immunizing SIR epidemic. The documentation of this smallpox epidemic and Cross’ accompanying analysis is a significant contribution of early epidemiological theory which deserves wider recognition.

The paper proceeds as follows. First, we give a brief background on the state of numbers in infectious disease studies prior to 1819. We then introduce John Cross along with a history of smallpox epidemics before 1819 in the city of Norwich, England. After, we use Cross’ data and observations to describe the smallpox epidemic of 1819. We then interpret these data in terms of geometric models (Brownlee’s Normal law [10] and Kermack–McKendrick’s square hyperbolic secant law [4]), SIR and SI^n^R differential equation models, and finally, Volz–Miller random-network models. Along the way, we show how Cross’ inclusion of auxiliary demographic data in addition to epidemic dynamics data greatly improves our ability to make inferences. Finally, we compare the disease attributes we infer from these models to attributes of smallpox used by modern smallpox modelers.

## Materials and methods

### Medical statistics before 1819

Cross’ work lies in a transitional phase in the history of the quantitative understanding of infectious diseases. Over the century and a half preceding 1819, quantification in public health and medicine had been developing along two complementary lines. The first was a drive in government to develop natural laws of the state—“political arithmetic”. This began with John Graunt’s “Natural and Political Observations … upon the Bills of Mortality”, which appeared in 1662 [17]. The subsequent progress in demography and the related development of statistical mathematics for data analysis eventually infiltrated the public-health movement in the first half of the 1800’s (*i.e*. [18]).

The second line was more medical. The introduction of variolation into England in 1721 by Lady Mary Wortley Montagu, and it’s subsequent promotion by Hans Sloane [19], forced physicians to consider more nuanced perspectives on preventative medicine. It was universally accepted that smallpox infection was potentially lethal [20]. However, variolation could also be lethal [20]. Thus, English physicians faced a difficult choice between a treatment that might kill an otherwise-healthy patient, or inaction that left the patient vulnerable to a disease that also might kill them. Some physicians believed that there was still a balance to be struck. For example, Hans Sloane is said to have explained to Princess Caroline that he could not recommend variolation on its own, but that the dangers of smallpox infection might be much worse than those of variolation [19]. But without the language of statistics and systematic data to support them, the debate had difficulty rising above randomized experiences of individual physicians. Fortunately, such a language was almost at hand. In 1722, James Jurin attempted to use data and calculation to demonstrate that in the environment of the day, the risks of variolation were greatly out-weighted by the risks of smallpox itself [21]. It was almost immediately pointed out by Isaac Massey (in the primitive language of the time) that the calculations could not be taken at face-value because access to variolation was positively correlated to economic status and the associated benefits that could improve outcomes post-infection [22]. Further data and calculations with regard to the issue were communicated over the next century by many others [17, 23] including statesman Benjamin Franklin [22] and mathematician Daniel Bernoulli [24, 25]. The debate receded with Jenner’s introduction of safer cowpox-based vaccination in the late 1700’s [26], but quantification had been firmly established as part of the smallpox conversation by that point.

By 1819, there was, at least among a few people, an understanding of (1) the value of demographic statistics, (2) the necessity of numbers and calculation in understanding certain healthcare trade-offs, and (3) the importance of collecting data to support statistics and calculation. But, despite numerous efforts [14], the syntheses of these ideas was still rather limited, and we have not yet found evidence that anybody had developed a complete self-contained statistical picture of an epidemic wave prior to 1819.

### Cross and Norwich smallpox

The author of the chronicle we use was a British surgeon named John Green Cross (1790–1850) [27]. Cross was born in 1790 to a farming family in Suffolk, England. Early on in his life, John was apprenticed to a surgeon-apothecary in Stowmarket, the daughter of whom he married in 1815. Upon conclusion of his apprenticeship, Cross traveled to London to study at St. George’s Hospital. After spending some time studying in Dublin and Paris, Cross settled in Norwich in March of 1815. He served as witness of the epidemic we study here, of which he published his account “A History of the Variolous Epidemic which occurred in Norwich in the year 1819” [13] in 1820. The book was favorably reviewed by the North American Review [28] and the Edinburgh Medical and Surgical Journal [29] at the time of publication. In 1823, Cross became assistant-surgeon to the Norfolk and Norwich Hospital, and became surgeon in 1826. Cross was awarded the Jacksonian prize at the College of Surgeons of England in 1833 for his work on “The Formation, Constituents, and Extraction of the Urinary Calculus.” Over his life, he mentored forty apprentices, one of which went on to be the first professor of surgery at Cambridge. John Cross died on June 9, 1850, and was buried in Norwich Cathedral [27]. After his death, his book was cited by Simon [30] and Creighton [14], but scarcely since then, with the notable exception of a popular history by Glynn and Glynn [20].

In his book, Cross provides a brief history of the prevalence of smallpox in the city of Norwich. This history suggests smallpox epidemics were sporadic occurrences in Norwich rather than annual events like the plague epidemics in Bombay. Cross begins with an epidemic from 1805, about which he says that beforehand smallpox had almost been extinct in Norwich for some time. From this we can infer that the city of Norwich had not been exposed to a major outbreak of smallpox for a relatively long period of time. The cause of such a drought is left unspecified. Shortly after this epidemic, Cross describes another outbreak which lasted from 1807–1809 and brought with it 203 recorded deaths due to smallpox. Cross sites the duration and destruction of this epidemic to be due to the portion of the population which had gone unvaccinated. The last outbreak to occur before the focus of Cross’ text occurred in 1813, and lasted from February to September, leading to the death of 65 individuals. After this epidemic, until the outbreak in 1819, Cross does not believe that more than a single person, who did not spread this infection, was infected with smallpox within the city. Those 6 years of absence primed the city for the epidemic on which we will now focus.

With insight that still rings true today, Cross identified a primary cause of the 1819 epidemic he presents as

The neglect of vaccination, it will be presently shown, was one of the reasons of the extensive prevalence of this pestilence (12).

Cross claims that three quarters of the susceptible population came down with the disease during the epidemic (pg. 13). Making an observation much before its time, Cross writes

It therefore remains to be accounted for, why the disease spread thus rapidly, expiring, as it were, of starvation, in a few months, whilst commonly it might have gone on for two or three years, before the same number of individuals, distributed amongst a population of 40,000 inhabitants, would all have taken it (13).

He identifies that this outbreak likely ended because it exhausted the susceptible population. This clearly fore-shadows our modern understanding, and precedes a similar conjecture by Hamer [31] by nearly a century.

Cross’ clear-eyed documentation of the epidemic is rather remarkable, given the state of science when he was writing. Most others chronicling epidemics, such as Boylston’s account of smallpox in Boston in 1724 [32] and those reviewed by Creighton [14], fail almost universally to synthesize their observations into useful tables and numbers the way Cross does. Cross’ clarity on the demographics of his city’s population is particularly notable, as such denominators can be vital in model fitting (as we will see momentarily), yet were seldom attended to by those focused on the practice of medicine.

### The Norwich data

First, in reference to possible issues of ethics, all individuals from whom the following data has been collected are dead, and the data is fully anonymized.

In 1819, in the city of Norwich, England, a smallpox epidemic occurred which spanned the entire year. Over the duration of this epidemic, the deaths of 530 individuals were attributed to smallpox infection. Cross traces the origin of this outbreak to a girl who was exposed in a market town during a journey from York to Norwich and broke out in smallpox upon arrival in Norwich. This index case occurred at the latter end of June, 1818. Cross states that “the earliest smallpox cases seen by any medical man could be traced to this origin.” He traces the infection from this origin through the handful of cases which occurred at the end of 1818, resulting in only 2 deaths, but he does not provide us with extensive data on the outbreak until January 1819. Because we are without data for this 6 month time period, it makes using this initial date difficult for fitting the model. The primary time series data which we utilize for model fitting is presented in Table 1, which is a recreation of the page 5 table of [13]. For our purposes, we re-express the important data in Table 2.

**Table 1:** Cross’ Norwich mortality data for 1819.

Month of 1819	Smallpox deaths	Other deaths	Total deaths
January	3	61	64
February	0	71	71
March	2	68	70
April	15	61	76
May	73	63	136
June	156	70	226
July	142	61	203
August	84	63	147
September	42	96	138
October	10	63	73
November	2	62	64
December	1	83	84
Total	530	822	1352

**Table 2:** Cross’ data from Table 1 is equivalent to the following timeseries.

Index (*i*)	Day of 1819 (*t*_*i*_)	Smallpox deaths accumulated (*D*_*i*_)
1	0	0
2	31	3
3	59	3
4	90	5
5	120	20
6	151	93
7	181	249
8	212	391
9	243	475
10	273	517
11	304	527
12	334	529
13	365	530

Along with this time-series data, Cross provides us with data on the age ranges of those that perished due to the disease which we recreate in Table 3. It is worth noting that of the 530 recorded deaths to smallpox, 477 were born after the 1813 smallpox outbreak which had the last mentioned cases of smallpox in the city. These numbers are consistent with the biology of smallpox, as those who have survived infection are imparted with immunity to further infection. Therefore, we expect that many of the susceptible individuals in the population will have been born after the last epidemic, which is certainly reflected in the death data. The models we study later in this paper do not consider age structure, but utilizing this data which Cross provides would be a logical next step for making more realistic models.

**Table 3:** Age distribution of smallpox deaths during 1819 epidemic.

Age	Smallpox deaths
Under 2	260
2 to 4	132
4 to 6	85
6 to 8	26
8 to 10	17
10 to 15	5
15 to 20	2
20 to 30	2
30 to 40	1

There is the potential that Cross’ has made errors in recording deaths, either mis-attributing them to smallpox, or not recording deaths which were caused by smallpox. The former is unlikely as smallpox infection is visually apparent during the eruptive phase, and the doctors at the time will have been familiar with diagnosing smallpox. The latter is a possibility, but we do not directly address it in our subsequent models. We leave it for future work whether a nuanced approach to handling missing data gives models which predict the data well while also cohering with smallpox biology.

### Population estimation

In fitting a model, one of the important questions is how many people were present in the city of Norwich in 1819, and how many of them were susceptible to smallpox infection. On page 13, Cross tangentially suggests the population of the city *N* ≈ 40, 000 souls at the time of the epidemic. This is consistent with his other comments, such as on page 33 where he says 10, 000 = *N*/4, and on page 6 where he suggests 3, 000 = *N*/13 (implying *N* ≈ 39, 000).

Other sources also provide population estimates for Norwich around 1820. Creighton [14] cites 50,000 as the population at the time. Edwards [33], in a survey of mortality bills up through 1830, reports a population census in Norwich of about 50, 000 in 1821, and based on interpolation through adjacent census periods, would suggest an estimate of 49, 000 in 1819. Although larger, they aren’t significantly different from Cross’ estimate, so for the remained of the paper we will utilize Cross’ 40, 000 person estimate.

A majority of the first two chapters of Cross’ book are spent discussing the state of vaccination and general immunity in Norwich leading up to and during the epidemic of 1819. Cross stresses the difficulty he and the other medical men had in convincing people, especially in the impoverished classes, to immunize their children during the period between 1813 and 1819 when smallpox was non-existent in the city (pgs. 20–21). He continues with a discussion of the significant effort undertaken during the epidemic to vaccinate those yet uninfected, including a paid incentive for those who received a cowpox vaccination of which Cross’ provides data for on page 28. Because of the nature of smallpox, it is generally expected that someone who had been infected in a past outbreak, had been inoculated with smallpox, or had received the cowpox vaccine would be immune to further infection. Cross spends some pages discussing seeming exceptions to this general rule, and how the occasional failure of the cowpox vaccine complicated the vaccination effort (pg. 25). Norwich was also subject to a massive demographic shift during this period due to the rapid urbanization of rural populations facilitated by the industrial revolution (pgs. 12–13). These recent arrivals were less likely to be exposed to smallpox historically, while also being the mechanism by which Cross determines smallpox originally was introduced to the city in the summer of 1818.

How can we make sense of the many factors playing into the size of the susceptible population and come to a concrete number? Cross, while providing us all the context expounded above, also provides on page 7 data from his personal experiences with affected families during the height of the epidemic in 1819. From the beginning of March to mid August 1819, Cross attended to 200 cases of smallpox, from 112 families which totaled 603 people. Of the people uninfected in 1819, 15 had no history of infection or vaccination and thus resisted it, 91 had been vaccinated, and 297 had been previously infected. This gives a rough proportion 1 in 3 people in an effected family got infected. If we presume these families are a representative Norwich as a whole, then coupled with Cross’ population estimate of 40,000 people this gives an estimated 13,333 people susceptible to smallpox.

This susceptible population estimate explicitly depends on the assumption that Cross’ population sample is representative of Norwich in totality. As Cross has said, this epidemic was more prominent in the impoverished classes, so this sample potentially has fewer affluent families than is representative of Norwich, although Cross gives no particular class information about the city. This sample also suffers from the bias of all the families having members infected with smallpox. It is reasonable to believe that Cross’ data is representative of families which were effected, but is unclear how representative this selection of 112 families is of the city’s population. What is apparent is the estimate of 13,333 susceptible people forms an upper bound for this value.

We take care in explicating the many factors and information we have regarding the state of immunity to smallpox in Norwich because knowledge of the susceptible population is necessary to discern the transmission rate when the total population is held constant, which we assume for Norwich during the year of the epidemic. This estimate which we extrapolate from Cross’ data limits the total susceptible population to a realistic value given the history of smallpox and pox vaccination in Norwich at the time of the epidemic while also providing a fixed, informed value to then infer transmission from.

We also use Cross’ estimate of 1/6 from page 6 as the probability of mortality for infected individuals to simplify the fitting process. This value is directly inferred by Cross from his personal experience treating 200 cases, of which 46 terminated with death, along with the 530 smallpox deaths he recorded over the duration of the epidemic of which he says had infected “considerably above 3000 individuals.” With the exception of a single model, we utilize this probability to further reduce the number of model parameters we have to fit to the data.

## Results

We now explore the interpretation of Cross’ smallpox data in Table 1, in light of classical modeling. Aside from Farr’s auto-regressive modeling [12], the earliest models were a Gaussian in the fashion of Evans and Brownlee [10], and a square hyperbolic secant following Kermack and McKendrick [4], which were early developments in mathematical epidemiological modeling. Then we discuss an SIR-type model, which reveals the shortcomings of using compartmental models to fit this data, and we present a model in the vein of the Volz and Miller [34–36] SIR extension. This final model rectifies the inferred attributes of the Norwich epidemic we obtain from Cross’ data with attributes of smallpox used in the wider literature. There are many more complex smallpox models one can fit to this data set, but as this paper is focused on providing a convenient historical example to ground standard SIR theory while also revealing its shortcomings and methods of remedy, we will focus solely on these simpler models.

To reiterate what we discussed above in more detail, unless otherwise stated, we use the susceptible population estimate of 13,333 which we derived from Cross’ estimate of the Norwich population as 40,000 and his direct observations of families afflicted with the disease. Although this isn’t an observation directly from Cross but a product of our inferences from his observations, we will occasionally refer to this value as “Cross’ *S*_0_” in the proceeding discussion. Because Cross only provides us with time series data for deaths, we need to fix the death rate, or the models will generally have more parameters than we can specify with the given data. Again we lean on Cross and his estimate of 1/6 of infected individuals dying to handle this issue. These two informed assumptions are sufficient for us to fit reasonable models to Cross’ data.

### Geometric models

The first significant developments of mathematical epidemiology emerged in the 19^th^ century, especially in the work of William Farr. His 1866 letter predicting the coming end of a rinderpest epidemic [12] is likely the first mathematically forecasted epidemic. He accomplished this by studying the trend of increase over four week periods, and noted that despite the number of active cases increasing over each period, the ratio between consecutive periods was decreasing. Today, we would classify his methods as auto-regressive time series analysis.

By the early 20^th^ century, the first attempts to interpret disease data in terms of models appeared in the work of John Brownlee [10]. Brownlee used a Normal distribution curve inspired by Farr to fit incidence counts. Two decades later, Kermack and McKendrick [4] presented the now ubiquitous SIR compartmental model. The computational barriers to numerically solving differential equations at the time made fitting such a model to their data untenable, so they instead derived a square hyperbolic secant approximation of death incidence where they chose parameters which gave the curve a good visual fit to the data. We now have much more powerful and precise computational tools for fitting these two models to data.

As we have presented, Cross provides monthly death incidence data presented in Table 1, from which we constructed the cumulative death time series data in Table 2. Kermack and McKendrick in [4] chose the coefficients of their square hyperbolic secant to closely reflect the trend in their deaths per week data, which we can emulate here against the deaths per month incidence data. On the other hand, we can integrate these two incidence functions, the Normal Eq 1 and the square hyperbolic secant Eq 2, and fit these integrals to the cumulative death data we have constructed. When we present the modern disease models later in this paper, we will fit them to the cumulative death data, so in part we have decided to fit the integrals of these incidence functions to be consistent with these later models instead of historically consistent with Kermack and McKendrick. The choice we have made is further justified by the reality that cumulative death data is less sensitive to error introduced by Cross’ coarse time scale of months than incidence data is.

To tie the historical development of mathematical epidemiology together with the historical data set of Cross which we are presenting, we now fit both of these curves to the dataset. Specifically, based on observed case mortality chance *m* = 1/6, we select total number of infections *a*, maximum rate of infection (infected per day) *r*, and day this maximum occurs *c* for the normal-law

$$\begin{array}{c} D_{\text{normal}}\left(t;a,r,c\right)=\frac{ma}{2}+r\int_{0}^{t-c}e^{-\pi {\left(\frac{ru}{ma}\right)}^{2}}du, \end{array}$$ (1)

and square-hyperbolic-secant-law

$$\begin{array}{c} D_{\text{sech2}}\left(t;a,r,c\right)=\frac{ma}{2}+r\int_{0}^{t-c}{\text{sech}}^{2}(\frac{2ru}{ma})du, \end{array}$$ (2)

to minimize the root mean-square errors

$$\begin{array}{c} {\text{Error}}_{\text{model}}=\sqrt{\frac{1}{13}\sum_{i=1}^{13}(D_{\text{model}}\left(t_{i}\right)-D_{i}{)}^{2}} \end{array}$$ (3)

based on the data of Table 2.

**Fig 1:**
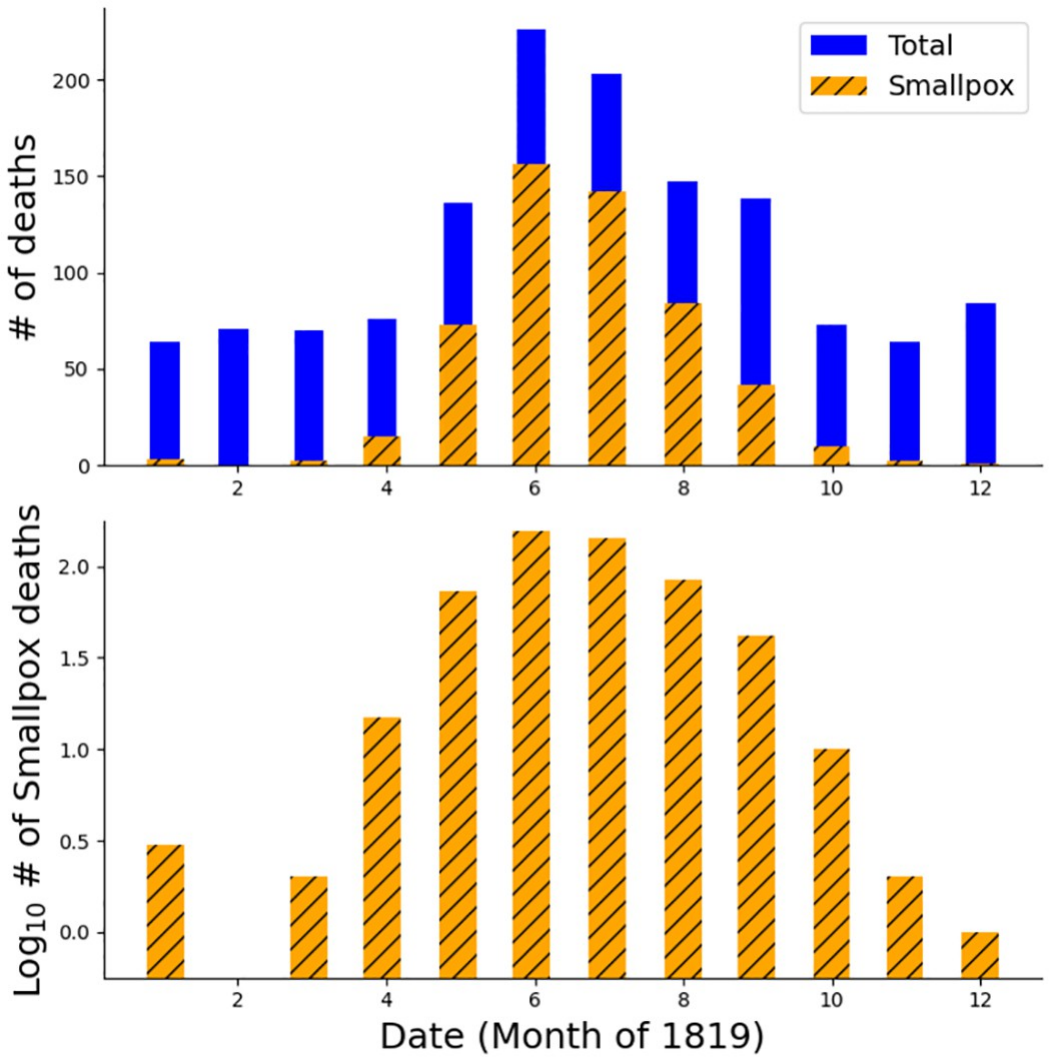
Smallpox mortality data. Plots of Cross’ 1819 Norwich mortality data (Table 1) on arithmetic (top) and logarithmic (bottom) scales. Note that the curves are not symmetric around their peak, but rather rise more quickly than they decline.

**Fig 2:**
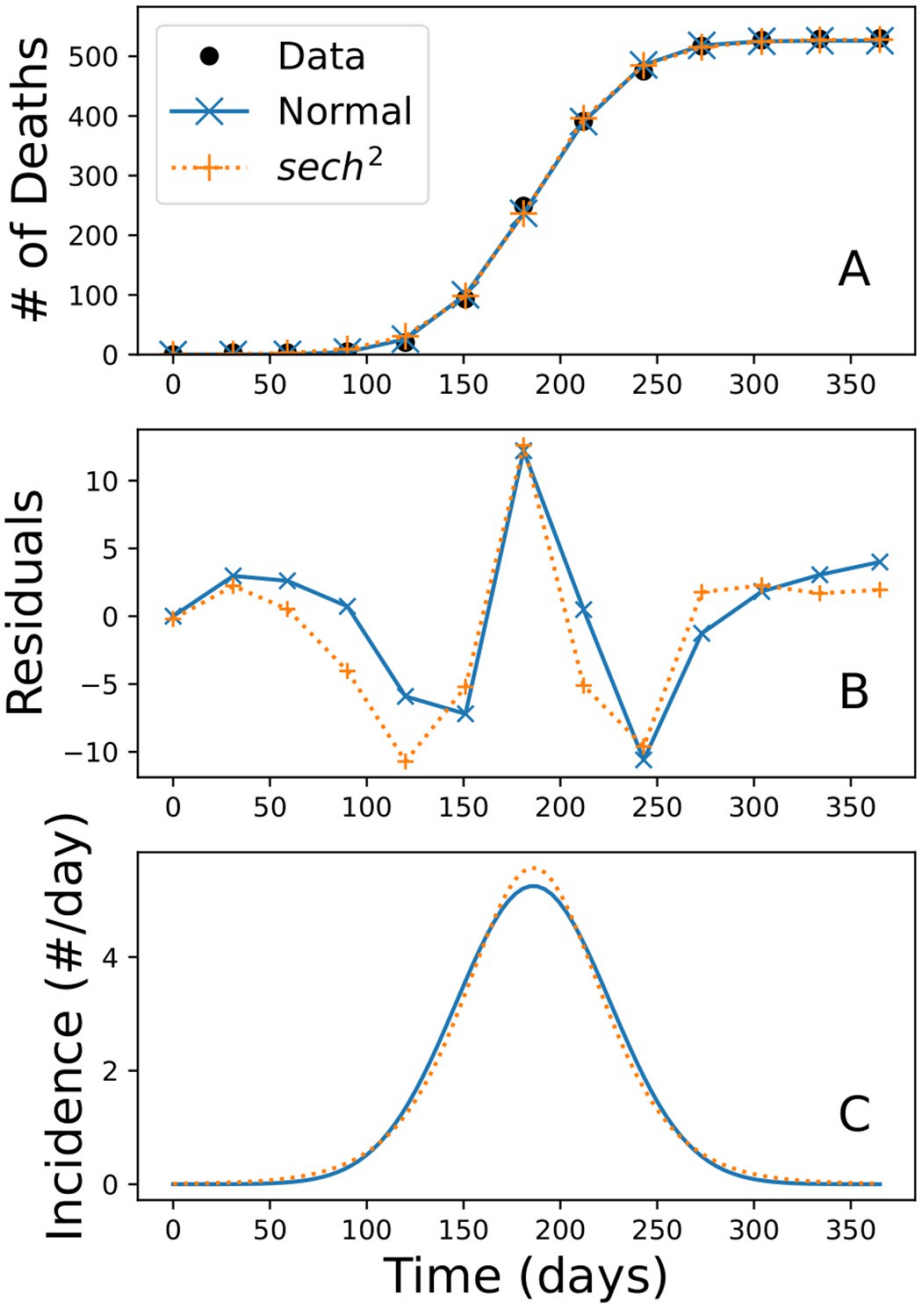
Classical geometric models. (A) Cumulative death curves for the normal-law and sech^2^-law minimizing Eq 3, (B) Residuals for each fit, (C) Projected incidence rates. Observe that both models predict symmetric patterns in incidence, but show oscillations in their residuals.

The specific parameters and details of our fitted equations can be found in Table 4 and Fig 2. Both fit the data reasonably well, with root mean-square errors of less than 6 cases. However, both exhibit systematic differences from the data because implicitly the assumed incidence rates are time-reversible. As Fig 1 shows, the principle increase in cases was faster than the decline in cases. The inability of these models to capture the asymmetry is best seen in the residual plot in Fig 2, where the residuals themselves are asymmetric, and reveal that the Normal model fits the first half better than the second, while the sech^2^ is the opposite. We do observe that both models are similar in their incidence rates up until the time around the peak, where the sech^2^ predicts a faster rate, and then they converge again for the remainder.

**Table 4:** Errors and best-fit parameter estimates for Brownlee’s normal-law and the Kermack–McKendrick square-hyperbolic-secant-law. Error is in deaths, *a* is the total number of infected people, *r* is the maximum rate of infection (infected per day), and *c* is the day this maximum occurs.

Model	Normal law	Sech^2^ law
Error	5.5	5.9
*a*	3,157	3,170
*r*	5.047	5.574
*c*	186	186

### SIR theory

Kermack and McKendrick’s SIR theory is a mechanistic epidemic theory that categorizes people based on their disease-state, and describes the expected dynamics of the counts in each category, based on simple principles of population mixing, person-to-person transmission, and elementary disease progression. It is a synthesis of preceding research by Ross, Hamer, Lotka, and Hudson. The simplest form of SIR theory is the compartmental SIR model, which is a system of three ordinary differential equations tracking the number of susceptible (*S*), infected (*I*), and removed (*R*) individuals under the hypotheses that the population is sufficiently strongly mixed for the law of mass-action to apply, that individuals can transmit the disease immediately after infection, and that the duration of infectiousness is exponentially distributed. To align the SIR model with Cross’ data, we will split the removed compartment into recovered *R* and dead *D*. The rates of change of numbers in each compartment are governed by the system

$$\begin{array}{c} \dot{S}=-\beta SI, \end{array}$$ (4a)

$$\begin{array}{c} \dot{I}=\beta SI-\gamma I, \end{array}$$ (4b)

$$\begin{array}{c} \dot{R}=\left(1-m\right)\gamma I, \end{array}$$ (4c)

$$\begin{array}{c} \dot{D}=m\gamma I, \end{array}$$ (4d)

where *β* is the transmission rate, 1/*γ* is the expected duration of infectiousness, and *m* is the probability of mortality. For those unfamiliar with Newtonian derivative notation $\dot{S}=\frac{d}{dt}S$ is the time derivative. These are solved from the initial condition (*S*_0_, *I*_0_, 0, 0). A key quantity in SIR theory and our general understanding of epidemics is the basic reproductive number $R_{0}$, defined as the expected number of infections generated in a naïve population from a single infected individual. In these SIR models, $R_{0}=\frac{\beta }{\gamma }N$, but in practical application when *S*_0_ and *N* are significantly distinct as Cross claims is the state of Norwich, $R_{e}=\frac{\beta }{\gamma }S_{0}$ more is reflective of the reality of smallpox early in the epidemic.

Given Cross’ own estimates of an initial susceptible population *S*_0_ = 13, 333 and a probability of mortality *m* = 1/6, the simple compartmental SIR model has 3 free parameters: $R_{0}$, *γ*, and *I*_0_. Estimating these parameters is roughly equivalent to estimating the initial growth rate, the duration of the epidemic, and the start-time. The estimates that minimize the root mean-square error are shown in the first column of Table 5. The fit is slightly better than those obtained by the geometric models discussed above. However, the parameter estimates themselves are not consistent with those described in other sources like Costantino et al. [37] (see Table 7). The basic reproductive number is too small and the duration of infectiousness is much too short.

**Table 5:** Errors and best-fit parameters for the three SIR variants we present. Units on *β* are people^-1^ × days^-1^, and of 1/*γ* is days.

Model	Cross’ Parameters	Best-fit *S*_0_	Best-fit *m*
Error	5.18	2.70	2.70
$R_{0}$	3.426	22.69	6.513
$R_{e}$	1.142	2.171	2.171
*m*	0.167	0.167	0.048
*S*_0_	13,333	3,827.12	13,333
*I*_0_	0.137	0.18	0.627
*β*	2.65 × 10^−5^	2.74 × 10^−5^	7.86 × 10^−6^
1/*γ*	3.24	20.83	20.83

**Fig 3:**
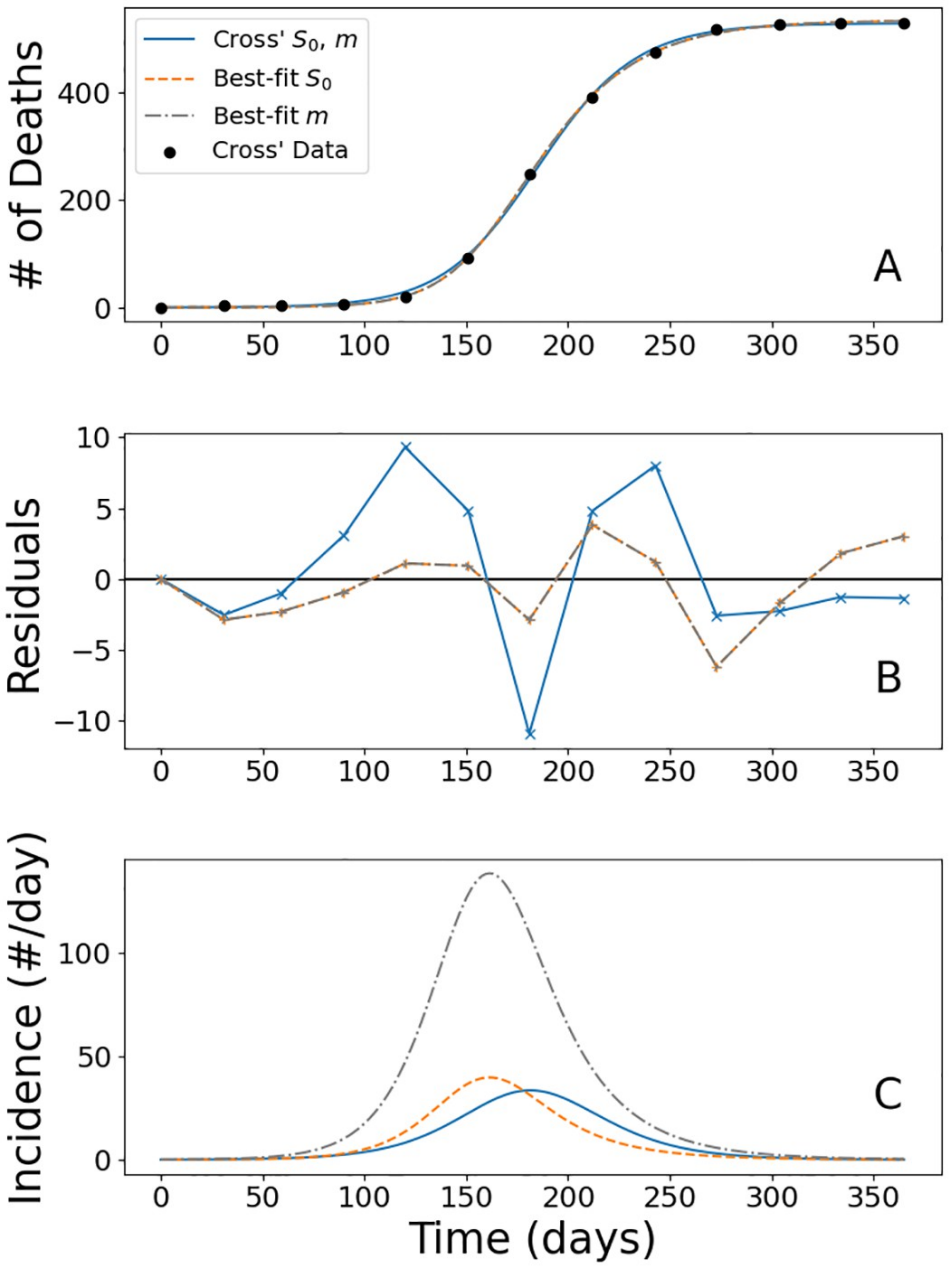
(A) This plot compares the SIR model with Cross’ *S*_0_ versus the best-fit *S*_0_ and *m* along side Cross’ data. (B) The residuals of the two best-fit models are here indistinguishable, and the performance is much improved during the height of the epidemic. (C) Here the incidence, or rate of infection, of the three models are presented. We observe the best-fit *m* has a much larger incidence due to the larger number of people infected during the epidemic. A structural difference is both best-fit models have an earlier peak incidence, while the peak incidence of the Cross’ model aligns more with the function models.

What is the source of the discrepancy between the estimated parameter values and those used by modern studies? Of the information we have derived from Cross’ account of the epidemic, it is possible that either Cross’ directly observed probability of mortality *m* = 1/6 or the initial susceptible population *S*_0_ we inferred is responsible. To investigate, we fit two additional SIR models, the first determining the best-fit initially susceptible population *S*_0_ while keeping *m* as originally specified, and the second the best-fit probability of mortality *m* while keeping *S*_0_ as we inferred. The details of each model are contained in Table 5, and the graphics are in Fig 3.

Looking at the errors, we see the model solely utilizing Cross’ estimates is an improvement over the two integrated function models, but its performance is much closer to those models than the two subsequent SIR models, which are a two-fold improvement. One attribute which is inferable from the parameters in Table 5 is the infectious period, which is captured in the quantity $\frac{1}{\gamma }$. For the first SIR model, this is an infectious period of 3.23 days, while the two best-fit models have infectious periods of 20.71 days, much more inline with the expected duration of a smallpox infection. The models also predict the number of total infections which occurred during the epidemic, of which the predictions are respectively 3,172 people, 3,198 people, and 11,142 people. Cross’ estimate that at least 3,000 people were infected conforms nicely with first two models, but his use of “considerably more” in describing how many infections did occur does not rule out the third model’s prediction explicitly. The first two totals of infection being around 3,000 is unsurprising since the total number of deaths Cross recorded was 530, and a fixed death rate of 1 in 6 necessitates that the model should predict around 3,180 total infections which both models align with. Cross’ speculates that the epidemic ends due to the exhaustion of the susceptible population, which the two best-fit models conform to with around 84% of the population getting infected under both models, while the original SIR model predicts only around 24% of the susceptible population suffering infection.

Studying Fig 3, especially plots A and B, along with the Error and $R_{e}$ in Table 5, suggest that the two best-fit SIR models are essentially the same regarding the data, even though they have entirely different parameters due to their different *S*_0_ and *m*, and Plot C reveals this distinction. What is occurring here is *S*_0_ and *m* are confounding in the form of the quantity *mS*_0_, which is approximately 2,222 for the first model using the estimates of *S*_0_ and *m* from Cross’ text, while for the two best-fit models *mS*_0_ ≈ 638. For such an SIR model and an *a* > 0, the parameters (*aS*_0_, *aI*_0_, *β*/*a*, *m*/*a*) give the same number of deaths, but not the same number of infections. Thus, with only death data, we can only determine the best-fit *mS*_0_ and the family models conforming to that. This analysis informs that there is no point in determining the best-fit model treating both *S*_0_ and *m* as free parameters, as they are tied together and only the quantity *mS*_0_ is identifiable within our data.

The nearly two-fold improvement in performance of these best-fit SIR models naïvely suggests that the inferences we made from Cross’ text incorrectly estimate *mS*_0_ despite these coming from his direct observations. One could speculate as to which quantity of *S*_0_ and *m* is the primary culprit, where the two models we fit presume either one or the other is a correct quantity, but this speculation is spurious. Yet, the results of these best-fit SIR models need not undermine Cross’ work or our inferences from it.

### SIR generalization and limitation

A natural next step for improving the SIR-type models is to expand the infection state to more compartments that better reflect the stages of infection. For smallpox, we should separate the infectious stage of our the SIR model into three separate stages– an incubative stage with no force of infection, a prodrome stage with a reduced force of infection, and the eruptive stage with full force of infection. As Cross only provides death incidence data, we argue that any expansion of these models, although biologically grounded, does not lead to further numerical precision. In fact, expanding these models will give durations of each stage of the infection without having any data to build that inference upon. All that can be inferred in terms of these durations is the total duration of being infected, in our models above $\frac{1}{\gamma }$, and this total will be segmented into however many infected compartments one would construct.

To further buttress this argument, we turn to computing the final size of an epidemic. Ma and Earn [38] demonstrate that the Kermack and McKendrick final size formula

$$\begin{array}{c} Z=1-\text{exp}\left(-R_{e}Z\right), \end{array}$$ (5)

where *Z* is the proportional final size, extends beyond the simple SIR model to an SIR-type compartmental model with any number of latent and infectious stages. This implies that extending the SIR models we have fit in biologically reasonable ways does not alter the final size of the epidemic so long as $R_{0}$ remains unaffected.

To apply this formula to the SIR models we have presented we must use $R_{e}$ instead of $R_{0}$ to account for the large proportion of the population which is presumed not susceptible. As we have argued, these models fit an optimal *mS*_0_, and we observe that $R_{e}$ is equal for the two best-fit models as well. This suggests that any model with an optimal *mS*_0_ has an equivalent $R_{e}$, which can be readily observed by expressing the final size as the function $Z(R_{e})$, taking the function value to be the positive solution of Eq 5 between 0 and 1. The total number of deaths is then

$$\begin{array}{c} D=mS_{0}Z\left(R_{e}\right). \end{array}$$ (6)

As Cross’ data specifies *D*, *mS*_0_ and $R_{e}$ are linked through this relation.

As *mS*_0_ does not depend on the addition of new latent or infective compartments and *D* is determined by the data, we must have that $R_{e}$ is fixed upon an optimal value regardless of the number of additional compartments, as the final size formula remains valid for these extensions. Thus we can conclude that given only death data there is no further utility to be ascertained by extending the SIR models we have constructed to be more biologically reasonable.

A primary assumption of the SIR model which we cannot reconsider by reparameterization or adding further compartments is the contact structure inherent in the mass-action hypothesis. Assuming a well-mixed population allows for a certain extent of asymmetry in the solution curves, but it may not be sufficient to capture the asymmetry present in the data. To investigate this assumption, and support Cross’ work which model-based analysis has thrown doubt upon, we now appeal to dynamic state-based models with more general contact structures.

### Volz–Miller theory

One potential explanation for the discrepancies between Cross’ data and compartmental SIR theory is the strong-mixing hypothesis used to justify the mass-action description of disease transmission. Human populations are rarely strongly-mixed, and contact structure can have a significant influence on epidemic dynamics.

Relatively recent mathematical epidemiology research by Volz and Miller [34–36], has provided a powerful generalization of SIR theory which can account for some heterogeneous contact structures without the heavy burdens of spatial structure, explicit contact networks, or agent-based simulations. This theory is more mathematical than general SIR theory and has not received the same extensive attention, so we provide a brief introduction to the theory in S1 Appendix.

We assume that contacts for an individual are momentary and the number of such contacts in the population is distributed following a negative binomial distribution NB(*r*, *ω*), where we parametrize it by *ω* the mean number of contacts. The individual probabilities of this distribution are given by

P(k)=(k+r-1k)(1-rω+r)k(rω+r)r. (7)

To use the language of Volz and Miller, the model we implement is an actual degree mean field social heterogeneous model [34, 35]. As *r* → ∞, the negative binomial distribution converges to a Poisson(*ω*) distribution, and thus the following models we study converge to the SIR model under this same limit of *r* → ∞, which is established in [34].

To define the Volz–Miller system of equations, we will utilize the probability generating function (PGF) Ψ(*θ*) and its derivative Ψ′(*θ*), for the contact structure of the population. Following the definition in [36],

$$\begin{array}{c} \Psi \left(x\right)=\sum_{k=0}^{\infty }P\left(k\right)x^{k}, \end{array}$$ (8)

where P(k) is the probability that a node has degree *k* given in Eq 7. Our choice of the negative binomial distribution to model the contact structure proves convenient at this juncture, as the PGF of the negative binomial distribution has a closed form. Utilizing this, we can express Ψ(*θ*) as

$$\begin{array}{c} \Psi \left(\theta \right)=(\frac{r}{r+\omega (1-\theta )}{)}^{r}. \end{array}$$ (9)

With all the individual pieces in hand, the Volz–Miller formulation is given by the following two equations,

$$\begin{array}{c} \dot{\theta }=-\eta \theta +\eta \theta^{2}\frac{\Psi^{\prime }\left(\theta \right)}{\Psi^{\prime }\left(1\right)}-\gamma \theta \;\text{ln}\;\theta , \end{array}$$ (10a)

$$\begin{array}{c} \dot{\rho }=\gamma \left(1-\Psi \left(\theta \right)-\rho \right). \end{array}$$ (10b)

These equations have an implicit network encoded into them, so given a random test node *u*, *θ*(*t*) solving Eq 10 is the probability that *u* has not been transmitted to by a randomly chosen neighbor. *ρ* is the normalized version of the removed compartment *R* in the previous SIR models, which will be made explicit in a moment.

With solutions to Eq 10, we can calculate the familiar compartmental state variables *S*(*t*), *I*(*t*), *R*(*t*), and *D*(*t*) through the relations

$$\begin{array}{c} S\left(t\right)=u\Psi \left(\theta \left(t\right)\right),\;\text{where}\;u=S_{0}/\Psi \left(\theta_{0}\right), \end{array}$$ (11a)

$$\begin{array}{c} R(t)=u(1-m)\rho (t), \end{array}$$ (11b)

$$\begin{array}{c} D(t)=um\rho (t), \end{array}$$ (11c)

$$\begin{array}{c} I(t)=u-S(t)-R(t)-D(t). \end{array}$$ (11d)

#### Volz–Miller model fitting

To study whether the population of Norwich conforms with the strong mixing hypothesis, we built three Volz–Miller models with increasing degrees of freedom. As there are many parameters in these models, we present all in Table 6. The first model is intended to recover the results of our SIR model using Cross’ *S*_0_ = 13, 333. The formulation of Volz–Miller we use converges to an SIR model as *r* → ∞, as mentioned above and shown in [35]. For this model we choose *r* “large” (*r* = 10^5^), and observe that the Volz–Miller model is converging to the SIR.

**Table 6:** Volz–Miller fit parameters with the Cross’ parameter SIR model for comparison.

Model	Cross’ SIR Model	SIR Approx.	Best-fit Contacts	Best-fit Contacts and *S*_0_
Error	5.18	5.17	2.56	2.56
$R_{0}$	3.426	3.44	5.676	5.607
$R_{e}$	1.142	1.147	1.892	1.869
*S*_0_	13,333	13,333	13,333	13,871
*I*_0_	0.137	1.078	0.214	0.215
*η*	–	0.0052	0.0502	0.0605
1/*γ*	3.24	3.327	15.175	14.803
*r*	–	10^5^	0.832	0.691
*ω*	–	65.58	1.276	1.74

The second model we built still utilizes Cross’ *S*_0_, but now we take advantage of the Volz–Miller framework to adjust the contact structure to get a better fit model. We do so by letting the model fit *r*, and observe that the resulting best-fit has smaller error than the SIR models we have presented. Interestingly, the best-fit values of *r* = 0.832 and *ω* = 1.276 imply the population is much more sparsely connected than strong-mixing would imply.

For the third model, we again open to question Cross’ estimation of *S*_0_, but contrary to the reduction which the second SIR model made, *S*_0_ = 13, 871 with *r* = 0.691 and *ω* = 1.74. This model is a slight improvement, roughly a hundredth of a death, over the previous model, while *S*_0_ is only 4% larger than Cross’ estimation. Unlike the SIR model, where we must believe our inference from Cross’ account wrong to get a better fit model, the Volz–Miller framework reinforces this estimate as being accurate, if only a slight underestimation, with the contact structure of Norwich being much sparser than well-mixing assumes.

Comparing the residuals in Figs 3 with 4, we observe the structure of the SIR with best-fit *S*_0_ is similar to the residuals of the two best-fit VM models. This suggests that what the Volz–Miller framework accomplishes in reducing the contact rate by considering a sparser contact structure is similar to reducing *S*_0_ in SIR, which in effect reduces the contact rate as well. These VM models ultimately perform better than the SIR because they are more able to match the asymmetry in the data.

**Fig 4:**
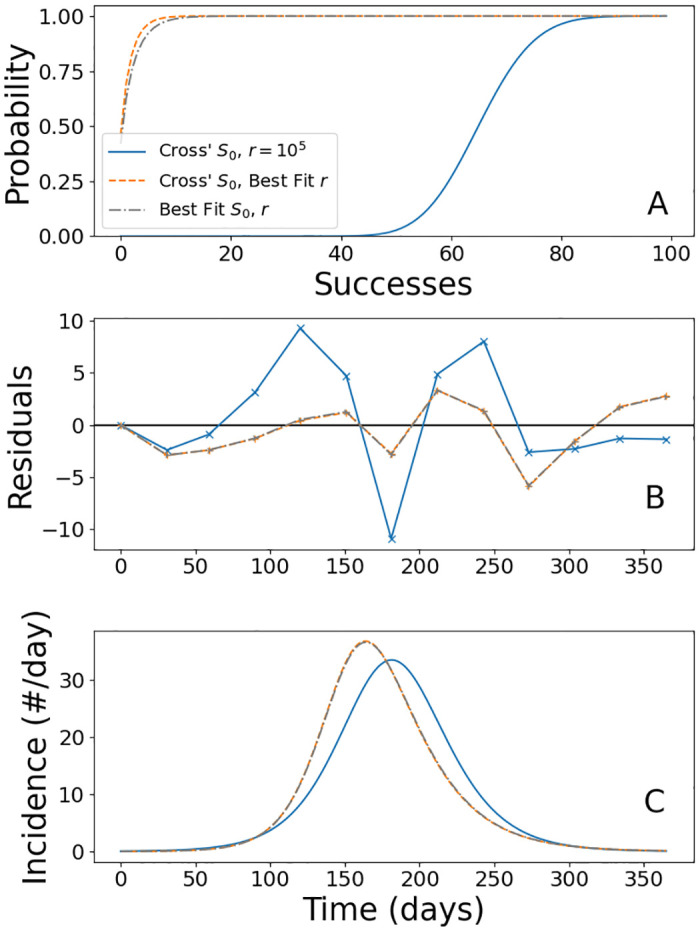
(A) The plot of the cumulative distribution functions for the three best-fit negative binomial distributions. (B) The residuals reveal that the mass-action approximation fits the data better at the tails, but is significantly worse than the other two models during the height of the epidemic. (C) The Incidence curve reveals the same trend as in the SIR models, where better fit models have an earlier peak incidence. The magnitude of these incidence curves are comparable to the SIR model with best-fit *S*_0_.

## Discussion

Cross’ work provides support for the idea that good modeling starts with a clear-idea understanding of the data, and that that data must be collated constructively—that it doesn’t just fall from the sky ready to use. Although there’s no sign in his writing that Cross was aspiring to anything more than pragmatic documentation of this epidemic, his exposition telegraphs systematic mechanistic thinking about the progression of an epidemic. He discusses Norwich’s history with smallpox, with an understanding that previous outbreaks have left residual immunity within the city. Cross describes the state of the city, and how the infection is introduced, sensing that what follows will depend in part on how things start. He also describes the course of events in both collective and individual terms, recognizing that neither is wholly adequate to an attentive reader when presented alone. His task is made somewhat easier by the particulars of smallpox, as opposed to harder-to-identify diseases like flu, and for that bit of luck, we can be grateful.

Farr is also a source of an historical smallpox dataset [39], but it does not contain time series data in the same fashion as Cross’ book. Instead, Farr focuses on general disease outcome and demographic data, which provides a broad view of smallpox in the 19^th^ century. Thus, Farr’s data is less useful for our modeling efforts, but does provide a view into what much of early epidemic data was like, and how enigmatic Cross’ data is, only adding to the appreciation for its existence.

One aspect of Cross’ text which we ignore is his discussion and data pertaining to the vaccination effort done during the epidemic. We make the assumption, after much labor, that the susceptible population at the start of epidemic is 13,333 people, and this population only depletes through infection. This leads to conclusions of our models which contradict statements made by Cross, like three quarters of the susceptible population being stricken by the disease (pg. 13) and the claim that after the epidemic, “not a hundred individuals were to be found in the city, who would be affected by exposure to the variolous contagion (pg. 15).” The best VM model we generate in terms of error has only 23% of the susceptible population becoming infected during the epidemic, and over 10,000 people are still susceptible in the end, much larger than Cross’ claimed 100.

What the best-fit VM models accomplish by greatly decreasing the connection of the implicit contact network may likewise be accomplished by building in a vaccination mechanism to the SIR models. Cross’ does provide data for number of the claimants of the monetary reward for cowpox vaccination, so this data could be utilized to fit a model. An interesting feature of this data is the general trend in cowpox vaccinations follows the trend of the epidemic, although the significant decrease in June gives the data a bimodal structure. Utilizing this data to fully capture all the mechanisms exhausting the susceptible class could generate models which fully cohere with Cross’ claims and descriptions, and this effort is left for future work.

### Comparison of models with other data

With the extinction of naturally occurring smallpox in 1980, parameter estimation has been relegated to utilization of historical data sets. Despite extinction, smallpox modeling is still important as it is a prime virus for bioterrorism. Since the early 2000’s there have been many studies which utilized a smallpox model with attempts at realistic parameters. Costantino et. at. [37] provides a very useful meta-study on the parameter choices from 42 smallpox specific modeling papers. For our purposes, this study provides values for durations of the different stages of smallpox infection with which to compare our fit parameters to.

The first major deviance in our approach is we don’t entirely account for the biology of smallpox in our model construction as we do not have a prodrome period, which many of the models surveyed in [37] included. Biologically, this is a period during infection where the infected is infectious, but less so than during the eruptive phase of the infection. One characterizing aspect of this period from a modeling standpoint is the prodrome period is typically parametrized with a lower infectious rate than the eruptive stage which follows, which could also be captured using a variable infectious rate during infection. An example of this is the model presented in Longini et. al. [40]. As we have argued, given our dataset, attempting to capture the precise biology of smallpox in an SIR framework is superfluous.

To compare our models with those surveyed by [37], we will compare the aspects of smallpox, such as infectious period, which can be inferred from the parameters we have obtained. One difficulty with this comparison is our models don’t distinguish between a latent period, prodrome period and infectious period, while Costantino et. al. provide estimates for all three. Our infection mechanism is best related to the eruptive phase primarily responsible for smallpox infections, so we will compare our values with the infectious period they provide. It is worth noting that of the 42 studies analyzed in [37], only two used actual historical smallpox data to inform their parameter choices. These two do not form outliers when compared with the other 40 studies, so we can presume that the sources of the parameter choices for the other studies are biologically sound without coming directly from data. We do recognize that accepting a measured value through collective agreement, coined intellectual phase lock by Luis Alvarez [41], has biased subsequent measurements of physical quantities like the charge of an electron [42], and potentially Ptolemy’s measurements of lunar eclipses [43], toward the accepted measurements and away from the later discerned more accurate measurement.

Table 7 compares the average values presented in [37] with the values determined from our three SIR models and three VM models. To be perfectly clear, these are the average values for parameter choices from the 42 studies [37] analyzed, not the averages from a set of data of smallpox infections. We observe our best-fit models have larger $R_{0}$, although the latter two VM models are approaching the Costantino et. al. value of 4.52 ± 0.68. The infectious period is similar, with our best-fit VM models only over predicting the 95% confidence interval of [37] by two days. If we add on the prodrome period to the Costantino et. al. value, which is 2.88 ± 0.14 days, this places our VM infectious periods nicely within the 95% confidence interval centered around an average of 14.96 days, although we caution that this could be cherry picking as we are deliberately ignoring the latent period which would add on another 11.92 ± 0.22 days to the total duration of the infection.

**Table 7:** Comparison between common smallpox values from [37] and inferred values from the models we have presented. Error bars on the Costantino et. al. values are a 95% confidence interval.

Model	Error	$R_{e}$	$R_{0}$	Infectious Period
Estimates from Costantino et. al. [37]	–	–	4.52 ± 0.68	12.08 ± 1.15
SIR with Cross’ *S*_0_, *m*	5.18	1.142	3.426	3.24
SIR with Best-fit *S*_0_	2.70	2.171	22.69	20.83
SIR with Best-fit *m*	2.70	2.171	6.513	20.83
VM with Cross’ *S*_0_, *r* = 10^5^	5.17	1.147	3.44	3.327
VM with Cross’ *S*_0_, Best-fit *r*	2.56	1.892	5.676	15.175
VM with Best-fit *S*_0_, *r*	2.56	1.869	5.607	14.803

The two best-fit Volz–Miller models we present do overestimate both the $R_{0}$ and the infectious period relative to those values presented in [37], if we take the latter to be the comparison in Table 7, but they are still not unreasonable for smallpox. These discrepancies ultimately could be due to limitations of inference in the mortality data Cross provides, differences in the biology of smallpox in the early 19^*th*^ century to the modern pathogens modeled by those models collected in [37], systematic errors in Cross’ data, or even an intellectual phase lock biasing the parameters. With our limited data, both from Cross’ and from smallpox outbreaks from the early 19^*th*^ century as a whole, we aren’t able to discern where these discrepancies primarily originate.

To directly address the veracity of Cross’ data, we built a simple SIR model following our first presented above to explore the possibility that Cross missed smallpox deaths at a uniform rate. We found through this model that Cross would have had to miss 75% of the deaths due to smallpox to make the disease attributes predicted by the model to match the attributes from [37], which would imply that at least half of the total deaths in the city would have gone unrecorded. This would be a significant discrepancy, and is difficult to believe, but a more careful approach to missing data might return more reasonable results. We leave that as an open question.

All told, we can be content that by coupling modern disease modeling methods like Volz–Miller with Cross’ immaculately presented but coarse deaths per month data, we can obtain estimates for both the $R_{0}$ and infectious period which are not out of place in Costantino et. al.’s dataset. There are still many factors of this epidemic to be explained, but what we have presented is a first step toward using mathematical models to better understand the facts on the ground in Norwich over 200 years ago.

## Conclusion

Cross’ clear-eyed documentation of the Norwich smallpox epidemic of 1819 is rather remarkable given the state of science when he was writing. Most others chronicling epidemics, such as Boylston’s account of smallpox in Boston in 1724 [32] and those reviewed by Creighton [14], fail almost universally to synthesize their observations into useful tables and numbers the way Cross does. Cross’ clarity on the demographics of his city’s population is particularly notable, as such denominator-factors can be vital in model fitting, yet were seldom attended to by those focused on the practice of medicine.

Over the years, a number of excellent papers have reviewed the history of mathematical epidemiology [44–50]. In this paper we are advertising an old and forgotten smallpox mortality data set consistent with and well-explained by SIR epidemic theory, especially the general contact-structure extension developed by Volz and Miller. Such an example was necessitated by the recurrence of the plague epidemic that Kermack-McKendrick originally utilized in their paper, and thankfully because of the diligence of John Cross over 200 years ago, we have a very simple data set from which to generate such an example. The smallpox epidemics in Norwich were not periodic, but true singular epidemics in the sense that the assumptions of standard SIR theory necessitate.

## Supporting information

S1 Appendix (PDF)
