# Supplementary material for: John Cross, epidemic theory, and mathematically modeling the Norwich smallpox epidemic of 1819
Source: PLoS One. 2024 Nov 13;19(11):e0312744. doi: 10.1371/journal.pone.0312744 (PMC11560002; doi:10.1371/journal.pone.0312744)
Supplement: S1 Appendix — (PDF) [file pone.0312744.s001.pdf]

Appendix A

Volz–Miller theory is an extension of the SIR model, replacing the mass-action mixing mechanism with a more general contact framework. [2, 4] present many approaches to generating models, of which we utilize the mean field social heterogeneity (MFSH) formulation of the actual degree models. This choice is made primarily because in the limit as contacts go to infinity, this model converges to the standard mass-action SIR. One of the key characteristics of the actual degree MFSH model is all nodes have a fixed number of neighbors determined by a chosen probability distribution but the precise relations between nodes are changing instantaneously.

To derive the equation, we consider three probabilities, Φ_S, Φ_I, Φ_R, which represent the probability a newly formed edge connects to a node in each compartment respectively. Then we can write

$$\dot{\theta} = -\eta\Phi_I = -\eta\theta + \eta\theta^2\frac{\Psi'(\theta)}{\Psi'(1)} - \gamma\theta\ln\theta \tag{12}$$

as the governing equation for $\theta(t)$. A full discussion of this formulation can be found in [3].

A key characterization of a disease which we want to discern from a model is $\mathscr{R}_0$. A mathematical introduction to $\mathscr{R}_0$ and methods to compute it can be found in [1]. For computing $\mathscr{R}_0$ for this model, we follow [3], with details of the exact calculation found in the supplement of that paper. We consider a newly infected node early in the epidemic, and want to compute how many direct infections are expected to arise from this single infected individual. This node has degree k with probability $\frac{k\mathbb{P}(k)}{\langle K\rangle}$, which is distinguished from selecting a node at random from the population whose probability

of degree k is $\mathbb{P}(k)$. The reason for this distinction is although the first infected individual in the population will have degree following the second distribution, all subsequent infections are more likely to happen to high degree individuals, so they follow the first distribution. Since the number of infections a node can cause depends on its degree, and the probability of degree of early infections is given by $\frac{k\mathbb{P}(k)}{\langle K \rangle}$, we will take this as the probability the selected node has degree k, and not $\mathbb{P}(k)$.

Now to compute $\mathscr{R}_0$, the probability that the newly infected node has degree k is $\frac{k\mathbb{P}(k)}{\langle K \rangle}$. Every moment this node has a new set of k neighbors, so there is no concern with keeping track of previous infections of neighbors, and since this is an early infection in the epidemic, we assume all k neighbors are susceptible during the full duration of the infection. Thus this node has an infection rate of ηk. The expected duration of an infection is then given by $\frac{1}{\gamma}$, so one would expect an individual to cause $\frac{\eta k}{\gamma}$ new infections. Averaging over the distribution of neighbors k then gives $\mathscr{R}_0$ to be

$$\mathscr{R}_0 = \sum_k \frac{k\mathbb{P}(k)}{\langle K \rangle} \frac{\eta k}{\gamma} = \frac{\eta}{\gamma} \sum_k \frac{k^2 \mathbb{P}(k)}{\langle K \rangle} = \frac{\eta}{\gamma} \frac{\langle K^2 \rangle}{\langle K \rangle} = \frac{\eta}{\gamma} \left(\frac{\Psi''(1)}{\Psi'(1)} + 1 \right).$$

For a more thorough introduction and discussion to these models, we point the reader to the works of Miller and Volz themselves [2–4].

References

1. van den Driessche P. Reproduction numbers of infectious disease models. Infectious Disease Modelling. 2017;2(3):288–303. doi:10.1016/j.idm.2017.06.002.

2. Miller JC, Volz EM. Model hierarchies in edge-based compartmental modeling for infectious disease spread. Journal of Mathematical Biology. 2013;67(4):869–899. doi:10.1007/s00285-012-0572-3.

3. Miller JC, Slim AC, Volz EM. Edge-based compartmental modelling for infectious disease spread. Journal of the Royal Society Interface. 2012;9(70):890–906. doi:10.1098/rsif.2011.0403.

4. Miller JC, Volz EM. Incorporating disease and population structure into models of SIR disease in contact networks. PloS one. 2013;8(8):1–14. doi:10.1371/journal.pone.0069162.
